# Toward Real-Time Detection of Intravenous Infiltration Using a Low-Cost Strain-Sensing Patch: Validation in Animal Models and Pediatric Clinical Monitoring

**DOI:** 10.1109/JTEHM.2026.3729926

**Published:** 2026-09-01

**Authors:** T. El-Khalili, S. Morgenstern, J. Fackler, R. Yang

**Affiliations:** Johns Hopkins University Baltimore MD 21218 USA; Yale School of Medicine New Haven CT 06510 USA; Department of Pediatric NursingCharlotte R. Bloomberg Children’s CenterJohns Hopkins Hospital Baltimore MD 21205 USA; Department of Anesthesiology and Critical Care MedicineJohns Hopkins University School of Medicine Baltimore MD 21205 USA; Department of Plastic and Reconstructive SurgeryJohns Hopkins University School of Medicine 1500 Baltimore MD 21205 USA

**Keywords:** Intravenous infiltration, signal processing, medical device monitoring, pediatric intensive care, strain sensor

## Abstract

Objective: To develop and evaluate a low-cost, skin-safe strain-sensing patch and real-time-capable algorithm for early detection of intravenous (IV) infiltration during continuous monitoring. Methods: A custom strain sensor composed of discrete piezoresistive elements was encased between two transparent medical dressings and mounted adjacent to the IV site. Data were collected during controlled IV infiltrations in juvenile pigs and during inpatient monitoring in pediatric patients without infiltrations. A strictly causal, event-based detection algorithm was implemented using features derived from the strain signal and its first difference. Performance was evaluated using event sensitivity, time-to-detection (TTD), and false-positive alerts per hour (FP/hr). Candidate thresholds were derived from the animal training-set evidence distribution. The operating point was selected using training-data sensitivity, detection time, and control false-alert burden, then fixed before held-out testing. Results: Across 16 animal recordings comprising 35 infiltration events, the algorithm detected 30 events (event sensitivity = 0.86) with a median TTD of 57 seconds at the selected operating threshold. Approximately 86.7% of detected events occurred within two minutes of infiltration onset. On the held-out recording subset, 8 of 9 infiltration events were detected, with a median TTD of 53.5 seconds. Evaluation on 117.8 hours of pediatric inpatient control data demonstrated a threshold-dependent false-positive alert burden, operating at approximately 1.2 alerts per hour at the selected operating threshold. Conclusion: A strain-sensing patch integrated with a real-time-capable, event-based detection algorithm enabled early IV infiltration detection at low infused volumes while maintaining a false-positive alert burden that is acceptable for early-stage clinical evaluation.

Clinical and Translational Impact Statement—Low-cost strain-based IV monitoring detected 30/35 animal infiltration events with a median time-to-detection of 57 seconds and approximately 1.2 false alerts per hour in pediatric patient monitoring.

## I. Introduction

Intravenous (IV) infiltration and extravasation occur when infused fluid escapes the vein and enters surrounding tissue, producing complications that range from localized swelling and phlebitis to more severe outcomes such as necrosis and compartment syndrome. The distinction between the two events is based on the infused substance: infiltration involves non-vesicant solutions, whereas extravasation refers to leakage of vesicant agents with higher potential for tissue damage [1].

These complications are common in pediatrics. In a prospective study of 151 peripheral venous lines in infants younger than one year, 58% infiltrated [2]. In a retrospective study of 1,174 hospitalized children, 92 infiltration events were documented (7.8%) [3]. Current monitoring relies on nurses to visually and physically assess IV sites for signs such as swelling and temperature change. Because such assessment is periodic rather than continuous, infiltration may develop between checks, and delayed recognition can permit progression to more serious injury [1], [3].

Multiple mechanical and infusion-related factors can precipitate infiltration or extravasation. Catheter dislodgment or vessel-wall puncture can allow the infusion to escape into surrounding tissue, while catheter location, securement, and properties of the infused solution can influence event risk and severity [4], [5].

Although pediatric infiltration has been recognized as a persistent challenge and interest in prevention has grown over the last two decades, particularly for neonatal patients, the number of devices available to clinicians remains very limited.

One category of devices in the literature consists of sensors that measure properties of the tissue around the cannula including strain, bioimpedance and optics [6], [7], [8], [9]. One of the most prominent commercial IV infiltration detection devices on the market falls into this category of sensors, ivWatch, which uses optical sensing and has demonstrated detection volumes as low as 0.2 mL, with an average detection volume of approximately 2 mL [7]. However, its cost structure may limit hospital-wide deployment because ivWatch requires a specialized proprietary monitor and an expensive single-use disposable sensor for each monitored IV site. The recurring per-site expense may restrict monitoring to selected high-risk patients rather than all patients with peripheral IVs, potentially reducing monitor utilization and making the capital investment more difficult to justify.

Another category of devices is designed to detect the same physiological signs that nurses evaluate at the bedside when monitoring for infiltration, including skin temperature, swelling, and tissue expansion. These signs typically become perceptible to human senses only in the mid-to-late stages of infiltration. Physiological sensors in this field have been evaluated in animal models, demonstrating low-volume detection before changes become readily apparent through visual inspection [8]. However, no devices in this category have made it to market. Although cost-effective, these devices are particularly susceptible to patient/nurse/parent motion artifacts that are common in the ICU environment. Because many of these sensors rely on adhesives for secure attachment, adhesive selection is also an important design consideration, particularly in pediatric intensive care patients where medical adhesive-related skin injury is a recognized concern [10].

The difficulties discussed for both categories of sensors likely play a large role in preventing more successful devices from being commercialized and widely used. However, it is unclear whether the methods of benchtop and validation testing commonly done in the literature are representative of a real infiltration, especially in the case of young pediatric and neonatal patients that receive infusions with low flow rates. Benchtop testing of both sensor types relies on the assumption that the extravasated fluid stays localized in an area around the sensor. While late-stage infiltrations are known to present with localized fluid accumulation, further testing is needed to understand the early dynamics of infiltration flow and accumulation across patient populations [11].

## II. Methods and Procedures

We chose to develop a device in the second category—one designed to detect the same physiological signs nurses observe, primarily skin strain/swelling and temperature. This approach offers a more cost-effective solution but comes with challenges such as increased noise due to motion artifacts and potential adhesive difficulties. To mitigate these drawbacks, we integrated our device into existing adhesives commonly used near IV sites, like a Tegaderm dressing [12], and implemented a detection algorithm capable of distinguishing between motion artifacts and true infiltrations.

### A. Skin Strain

During an infiltration, the skin and surrounding tissue at the IV site can expand for multiple reasons. Initially, fluid escaping the vessel wall seeps into the subcutaneous tissue, increasing pressure and stretching the skin. As the infiltration progresses, tissue damage activates an immune response, leading to blood vessel dilation, edema, and inflammation, which further contribute to skin expansion. To minimize tissue damage, the device must detect an infiltration at the onset of fluid leakage rather than responding to the secondary effects of edema and inflammation [4], [5].

Devices designed to detect skin stretching or swelling in the literature typically rely on strain gauges, load cells, or piezoresistive materials [6], [8], [11]. Strain gauges and load cells experience a change in electrical resistance in response to mechanical force, typically requiring secure, rigid attachment to a substrate using strong adhesives or industrial tapes. These systems are ideal for structural or industrial applications where firm mounting is feasible and necessary for accurate measurement. However, this level of adhesion may be unsuitable for direct application on human skin—especially in vulnerable populations like pediatric or neonatal patients—due to the risk of irritation or injury. In contrast, piezoresistive materials offer a more flexible alternative. These materials also change resistance in response to mechanical deformation but can be embedded into soft substrates such as medical dressings. This makes them particularly well-suited for skin-conformal biomedical applications, such as detecting localized swelling from IV infiltration, where gentle contact and adaptability to movement are essential [11].

Both types of mechanical deformation sensors must be securely mounted to accurately track skin deformation while ensuring that the force required to deform the sensor does not restrict natural skin expansion during swelling. Many devices have focused on improving sensor data fidelity by exploring different mounting methods—some attaching directly to the skin, while others are applied to flexible dressings commonly used on human skin. However, little research has examined how the force needed to deform these sensors changes based on mounting technique. If the force required for deformation is too large for a sensor mounted with an adhesive, it could limit the skin’s ability to expand during an infiltration. Therefore, it is crucial to select a sensor design that allows for secure mounting without interfering with the skin’s natural expansion.

To overcome the key limitations of existing mechanical deformation sensors in the literature, a custom-designed strain sensor was developed with two essential features. First, it consists of distinct piezoresistive segments that are in contact but not mechanically connected. Second, the sensor is enclosed between two layers of Tegaderm dressing (Fig. 1) [12]. Encasing the sensor between two layers of Tegaderm dressing provides several advantages. The first layer adheres directly to the patient’s skin, while the sensor is placed on top of it, followed by a second Tegaderm dressing layer that secures the sensor in place. This design eliminates the need for direct contact between the sensor and the patient while still ensuring accurate detection of skin deformation [12]. As the skin stretches, the bottom Tegaderm layer deforms accordingly, pulling the top layer along with it. Since the sensor is affixed to the top layer, it experiences the same deformation, allowing it to effectively measure skin strain [12].

**FIGURE 1.**
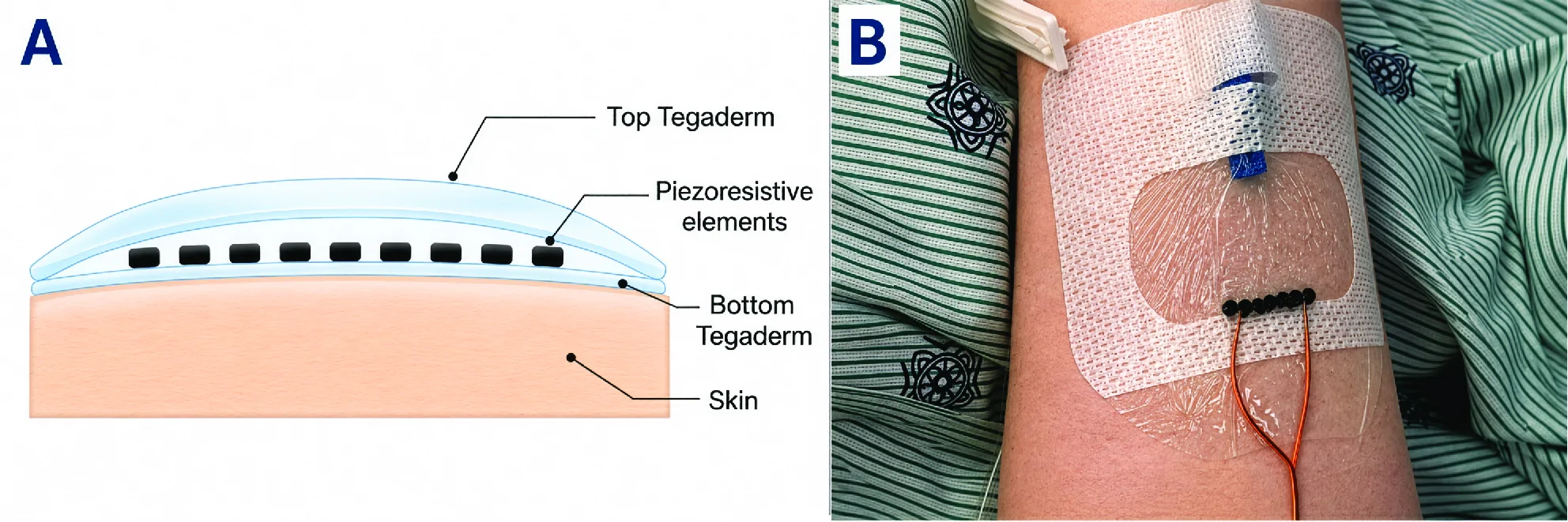
**Cross-sectional schematic and clinical placement of the IV infiltration sensor:** (A) Cross-sectional schematic of the custom strain sensor encased between two layers of Tegaderm dressing. The bottom Tegaderm adheres to the skin, the row of piezoresistive elements (black cylinders that are rectangles in cross section) deforms with tissue swelling, and the top Tegaderm secures the sensor. (B) Clinical photograph of the sensor in use on a pediatric patient’s forearm, demonstrating real-world placement adjacent to a standard IV site. Note that the thermistors used in animal testing are not shown here, as temperature data were not collected during the clinical study after animal trials revealed no significant thermal changes.

Tegaderm was selected for this application because it is a widely used, strong, and generally safe adhesive that conforms well to skin movement, thereby enhancing the fidelity of strain measurements [12]. Tegaderm is well suited for this application, but for patients who cannot tolerate the adhesive, the sensor can instead be configured between two layers of alternative hypoallergenic transparent dressings (e.g., Mepore, IV3000).

Many conventional IV infiltration detection sensors in the literature consist of connected piezoresistive elements arranged in various matrix or grid configurations. In contrast, our sensor features multiple discrete piezoresistive segments that remain in contact with each other, forming a continuous circuit, but are not connected, i.e., can be easily separated. At baseline, these segments are slightly compressed together and are fully touching, forming a complete circuit. When the Tegaderm deforms, the segments are pulled apart, reducing the amount of contact and parallel compression and increasing electrical resistance. With sufficient stretching, the segments eventually separate completely, creating an open circuit and producing the maximum signal output [12].

Because the segments are not mechanically connected but only in contact, the force required to deform them remains no greater than the force needed to stretch the Tegaderm. Additionally, since each segment is piezoresistive, the sensor not only detects strain parallel to the skin but also increases in resistance when compressed along the axis perpendicular to the skin. This dual functionality allows the sensor to measure skin strain at any location that stretches the Tegaderm in addition to pressure changes due to circumferential or diffuse swelling. The device is therefore not limited to infiltrations where a visible bump forms directly under the sensor and can detect a wide range of infiltration profiles [12].

At rest, the resistance of the sensor is on the order of 1 k *Ω *, and at maximum deformation the segments are no longer touching, meaning the resistance is infinite. This creates a large dynamic range that is a function of how much deformation the Tegaderm and consequently the skin has experienced. Designing the sensor to detect Tegaderm stretching means that the sensor must be securely mounted to the Tegaderm, not the skin, which is not only simpler, but means that the sensor never comes into physical contact with a patient’s skin [12].

Most devices in the literature focus on detecting localized bumps, but it is unclear if the flow rates in the Pediatric Intensive Care Unit (PICU) and Neonatal Intensive Care Unit (NICU) are high enough to create bumps with enough force to deform a sensor early during an infiltration. In these patient populations, high tissue malleability allows fluid to disperse over a broader area before becoming detectable, making localized bump formation less likely [11].

### B. Temperature Sensing

Temperature sensing is a commonly explored modality for IV infiltration detection, as changes in local skin temperature, similar to swelling, are among the earliest signs identified by bedside clinicians [5]. During infiltration, temperature changes may arise from extravasation of infused fluids that differ from body temperature or from local inflammatory responses following tissue irritation [5].

However, the utility of temperature as an early detection signal is uncertain. Infusions involving temperature-modified fluids represent a limited subset of clinical scenarios, and temperature changes driven by inflammatory responses may occur only after significant tissue injury has already developed. As a result, temperature changes may lag behind mechanical indicators such as tissue swelling or strain, limiting their effectiveness for early detection.

Temperature sensing was therefore evaluated in this study as a complementary modality rather than a primary detection signal. In this study, temperature data were collected during the animal testing phase to assess whether measurable temperature changes occurred during infiltration and whether they tracked the onset and progression of strain-based deformation. However, no consistent or clinically meaningful temperature trends were observed within the early detection window. Accordingly, temperature sensing was not incorporated into the final detection algorithm or clinical study phase.

### C. Device Construction and Schematic

The strain/swelling sensor functions as one resistor on a leg of a Wheatstone bridge and is paired with a 1 k *Ω * resistor. The other leg contains a 1 k *Ω * resistor and a potentiometer that ranged from 0-5 k *Ω *. A potentiometer is necessary because after placement of the sensor patch, there is some initial deformation based on the topology of the IV site. In order to account for various IV locations and topology, the sensor must be calibrated by changing the resistance of the potentiometer until the voltage dividers match in each leg. The voltage from each divider feeds into a differential amplifier with a gain of 10 and the output of the amplifier feeds into a microcontroller (Fig. 2) [12].

**FIGURE 2.**
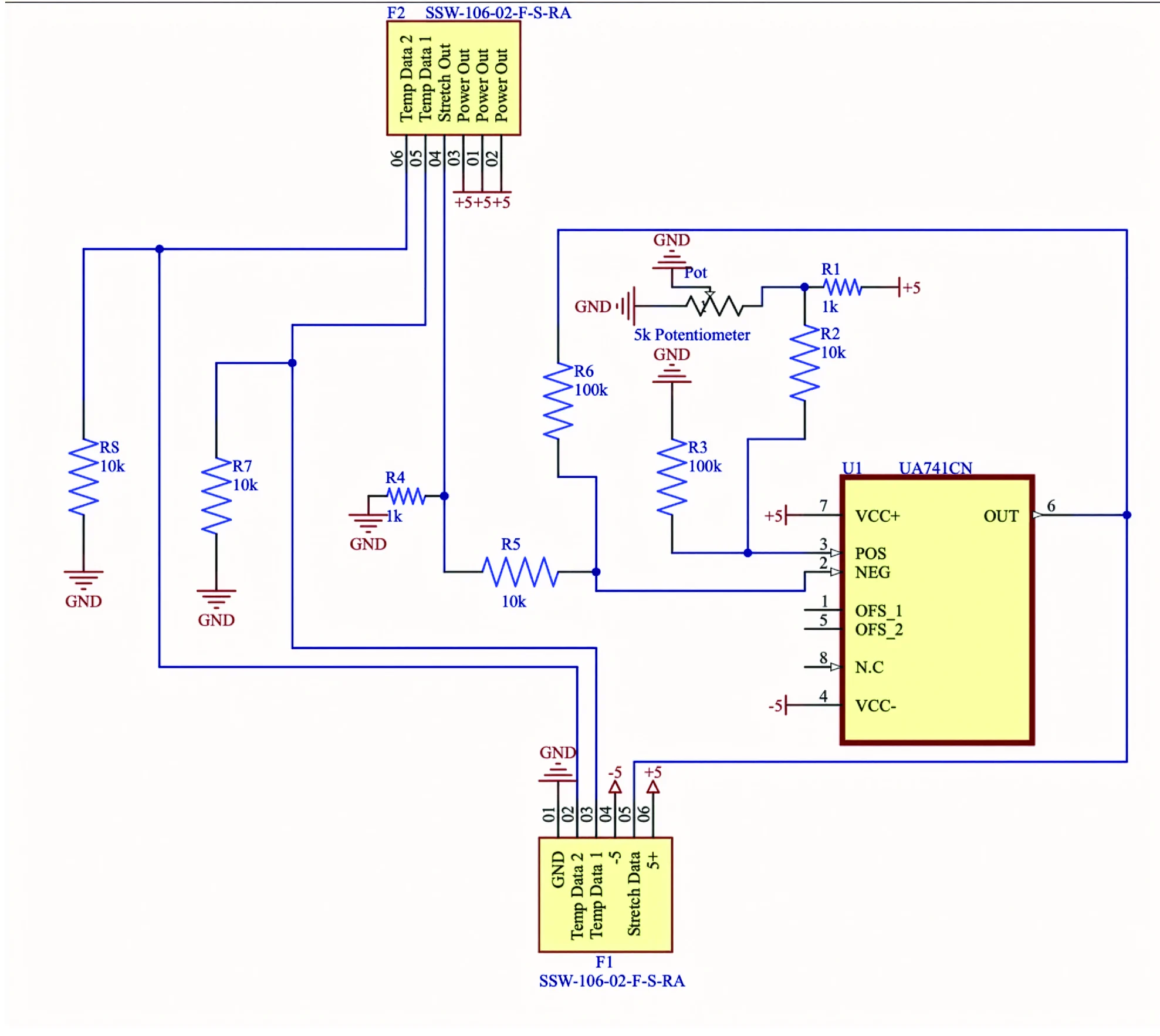
**Electrical schematic of the IV infiltration detection system.** Circuit diagram illustrating the signal processing architecture for the custom strain sensor. The sensor is configured as part of a Wheatstone bridge, with one leg containing the custom sensor and a 1 k *Ω * resistor, and the other leg comprising a 1 k *Ω * resistor and a 5 k *Ω * potentiometer for calibration. The differential voltage output is amplified by a UA741 operational amplifier and routed to a microcontroller. Additional voltage divider circuits (10 k *Ω * pull-down resistors) support optional thermistor-based temperature sensing, though these were not used in the clinical study due to limited effectiveness observed during animal testing. Power and data lines are routed through F1 and F2 connectors for integration with the external device housing.

To measure temperature during an infiltration, two thermistors (North Star Sensors 4AT103R44T2A) were positioned on either side of the custom strain sensor, which itself was standardized to ~2 cm from the IV catheter insertion site (needle entry point). In this setup, a proximal thermistor is located a short distance further up the arm relative to the strain sensor, while a distal thermistor is placed down the arm or hand. This configuration enables detection of localized temperature changes near the infusion site (Fig. 3) [12].

**FIGURE 3.**
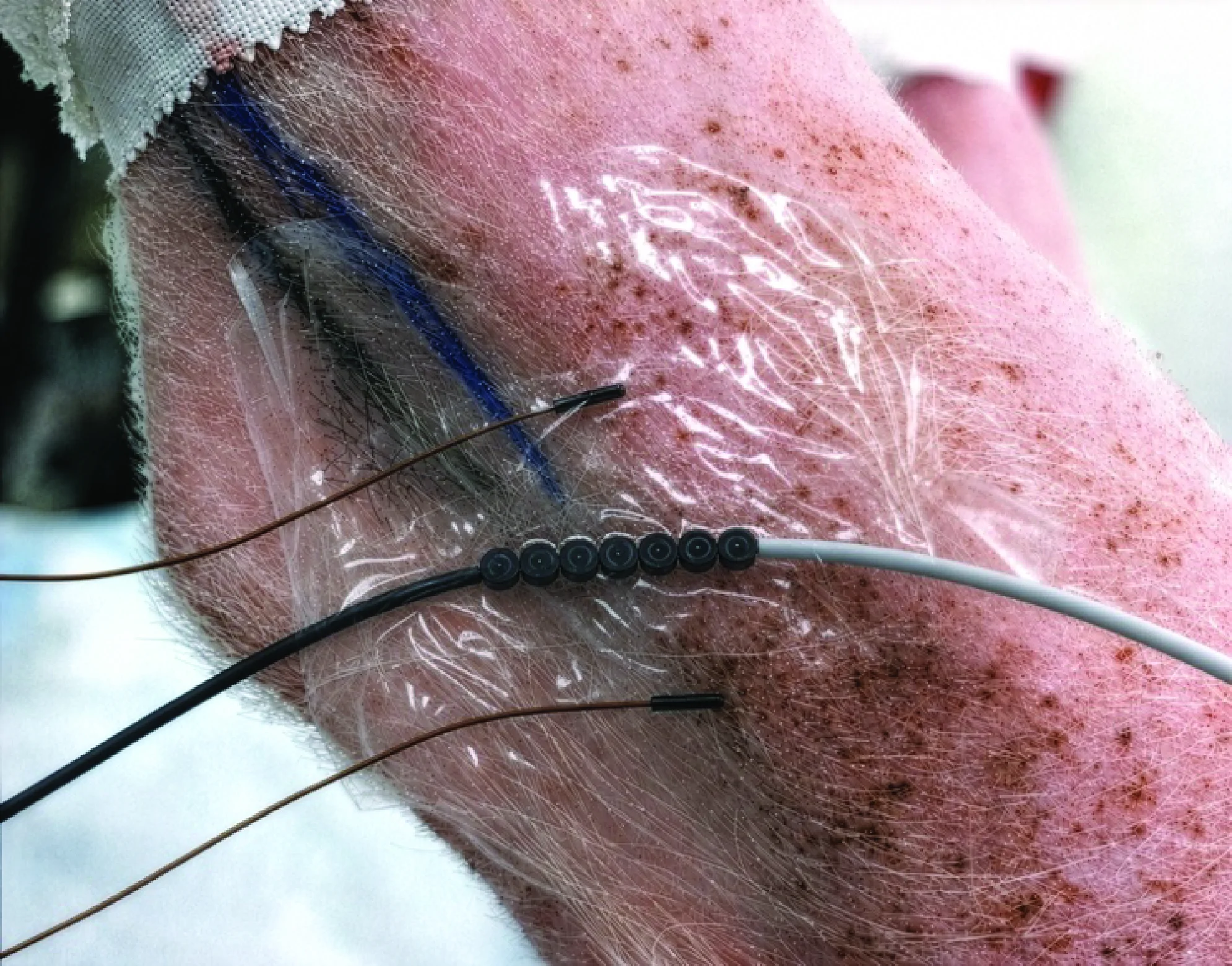
**Thermistor placement in the animal model.** Photograph from the piglet infiltration study showing the custom strain sensor and dual thermistors secured under Tegaderm. The two thermistor leads are positioned proximal and distal to the IV insertion site, enabling measurement of localized temperature gradients during induced infiltration events.

If an infiltration occurs, extravasated fluid or localized inflammation may lead to a temperature differential between these two points. For example, if cool IV fluid leaks into the surrounding tissue, the proximal thermistor may register a sudden drop in temperature relative to the distal thermistor. Conversely, if an inflammatory response is triggered, the proximal site may warm. By continuously monitoring this temperature gradient, the device could detect deviations that may indicate an infiltration [12].

We selected the North Star Sensors 4AT103R44T2A thermistor due to its balance of size, accuracy, compatibility, and cost-effectiveness. This 10 k *Ω * NTC thermistor (curve 44) features a tight $\pm ~0.2~^{\circ }$C tolerance over 0– $70~^{\circ }$C and operates across a wide temperature range ( $- 40~^{\circ }$C to $125~^{\circ }$C), making it suitable for detecting subtle skin temperature changes. Its mini bead form factor with insulated leads allows low-profile placement beneath Tegaderm without compromising patient comfort. The analog output integrates easily with microcontrollers, and at approximately *$*3 each in bulk, the thermistor remains affordable for scalable deployment.

The system is designed with a reusable electronic module and a disposable sensing interface, allowing the sensing patch to be replaced between patients while maintaining the core hardware components.

### D. Algorithm Development

The detection algorithm (Fig. 4) was implemented as a strictly causal, sample-by-sample, event-based pipeline designed for future continuous bedside monitoring. It was a rule-based signal-processing algorithm rather than a statistical or machine-learning model. Parameters were selected heuristically and refined empirically during algorithm development to balance detection sensitivity, latency, and resistance to transient artifacts, and were fixed before held-out testing. Although the analysis was conducted retrospectively, every update used only the current and preceding samples. Thus, the study evaluates real-time-capable behavior using offline data but does not constitute prospective, hardware-integrated real-time validation.

**FIGURE 4.**
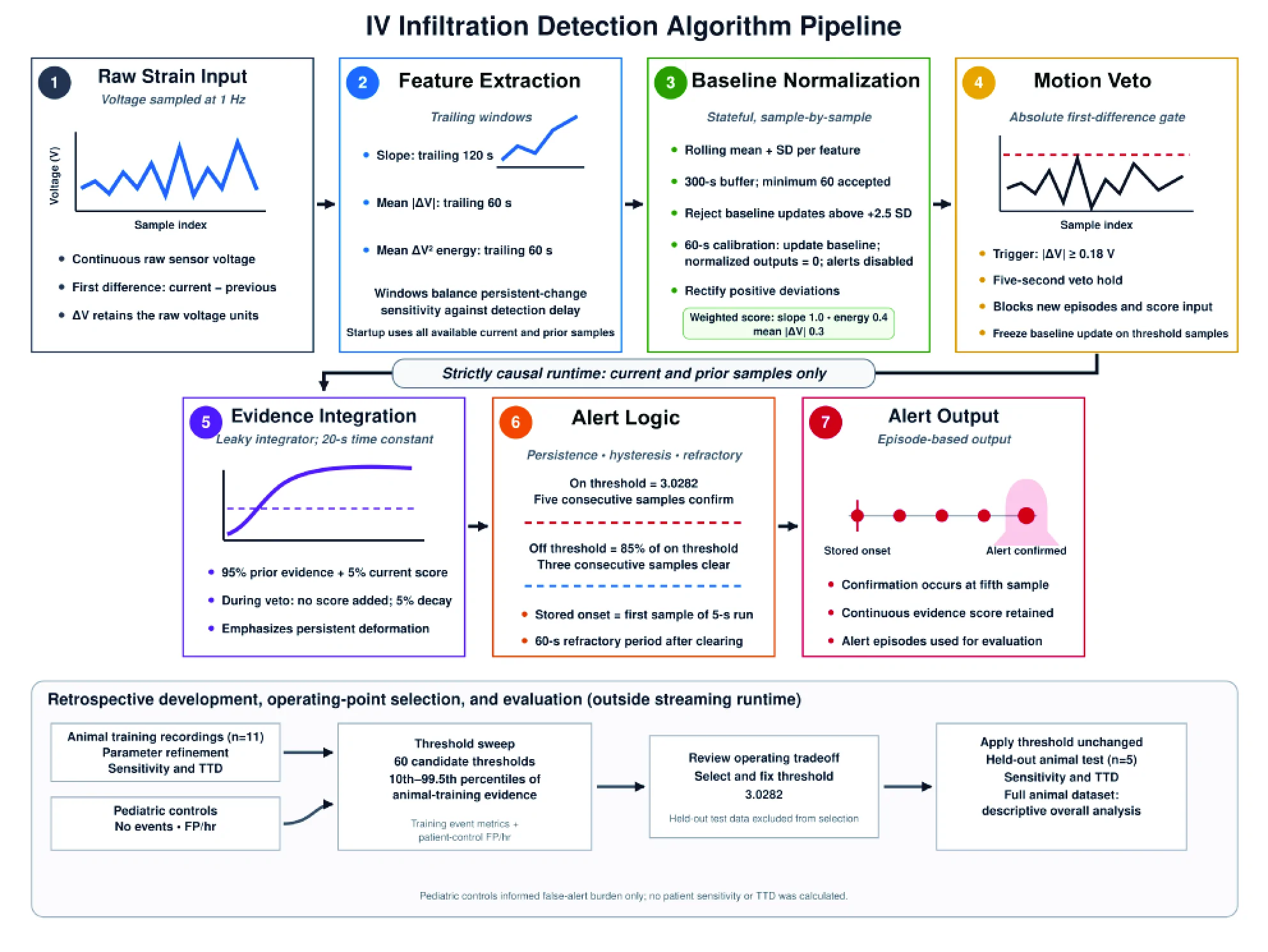
Overview of the IV infiltration detection algorithm pipeline. Raw strain sensor voltage is processed through causal feature extraction and baseline normalization with an initial calibration period. A motion/spike veto based on the absolute first difference suppresses transient artifacts, and a leaky integrator accumulates normalized evidence of sustained deformation. Alert episodes are generated using persistence-based thresholding with hysteresis and a refractory period. The lower panel summarizes the retrospective training-data threshold sweep, operating-point selection, and subsequent evaluation using the fixed threshold.

### 1) Stage 1: Raw Strain Input

Raw strain-sensor voltage was sampled at 1 Hz. The first difference was calculated by subtracting the immediately preceding sample from the current sample, producing a signal representing the sample-to-sample change in voltage.

### 2) Stage 2: Causal Feature Extraction

Three features were calculated using trailing windows: strain-signal slope over 120 seconds to capture sustained expansion, mean absolute voltage change over 60 seconds to characterize local variability, and mean squared first difference over 60 seconds to capture sustained higher-frequency activity. The window durations were selected empirically to balance sensitivity to persistent deformation against excessive smoothing and delayed detection. During startup, all available current and preceding samples were used until each window was filled.

### 3) Stage 3: Baseline Normalization & Calibration

Each feature was standardized independently relative to a continuously updated local baseline and rectified so that negative deviations did not contribute to detection. The normalized features were combined into an instantaneous score using fixed weights of 1.0 for slope, 0.4 for mean squared first difference, and 0.3 for mean absolute voltage change. These heuristic weights prioritized sustained tissue expansion while retaining complementary information from shorter-term variability. Baseline normalization used a 300-second rolling buffer for each feature, selected to provide a stable recent baseline while remaining responsive to gradual drift. At least 60 accepted samples were required before normalized values contributed to detection. Samples exceeding the current baseline mean by more than 2.5 standard deviations were excluded from baseline updates to prevent large upward departures from being absorbed into the baseline. During the initial 60-second calibration period, baseline updating continued, but normalized features were set to zero and alert initiation was disabled.

### 4) Stage 4: Motion/Spike Veto

A veto was initiated when the absolute sample-to-sample voltage change reached or exceeded 0.18 V and remained active for five seconds. These settings were selected empirically to suppress abrupt motion-related changes without unnecessarily delaying subsequent detection. During the veto, initiation of new alert episodes was blocked and the instantaneous score was not added to the evidence integrator. Baseline updating was also suspended on samples that directly met the 0.18 V criterion.

### 5) Stage 5: Evidence Integration

The instantaneous score was processed using a leaky integrator with a 20-second time constant, selected to emphasize persistent deformation while allowing brief fluctuations to decay. At each non-veto sample, the updated evidence retained 95% of the preceding evidence value and incorporated 5% of the current score. During veto samples, no current score was added, and the existing evidence decayed by 5% per sample.

### 6) Stage 6: Episode-Based Alert Logic

Five consecutive samples at or above the on threshold were required to confirm an alert episode. Once confirmed, the stored episode onset was assigned to the first sample of the qualifying five-sample run. An episode ended after three consecutive samples at or below an off threshold equal to 85% of the on threshold. This persistence and hysteresis reduced alerts caused by isolated threshold crossings or fluctuations near the threshold. A 60-second refractory period after episode termination prevented immediate re-triggering.

### 7) Stage 7: Alert Output

After the persistence requirement was satisfied, the algorithm generated an alert episode while retaining the continuous evidence score for ongoing monitoring. The alert was confirmed after the fifth qualifying sample, while the stored episode-onset time corresponded to the first sample of that run. Alert episodes, rather than individual above-threshold samples, were used as the primary unit of evaluation.

Algorithm development and evaluation used controlled animal recordings containing induced infiltrations with labeled event windows and pediatric inpatient recordings without infiltration events. Animal recordings were divided at the file level into training and held-out test sets using a fixed 70%/30% split. Because all animal recordings contained induced infiltration events, stratification by event presence was unnecessary, resulting in a random 70%/30% file-level split (11 training and five test recordings).

Infiltration events were defined as contiguous annotated response windows. An event was detected when the stored alert onset occurred between 10 seconds before the annotated event onset and the annotated event end. The 10-second early allowance accounted for annotation uncertainty and the possibility of measurable deformation immediately preceding the labeled onset. TTD was calculated from annotated event onset to the stored onset of the first qualifying alert. Consequently, accepted early detections could have TTD values as low as −10 seconds.

Alert episodes beginning outside all expanded event-matching intervals were classified as false-positive alerts. FP/hr was normalized by total non-event monitoring time. Event sensitivity was the proportion of annotated infiltration events detected, while event-level precision was the number of detected events divided by detected events plus false-alert episodes. Pooled pointwise area under the receiver operating characteristic curve was also calculated using sample-level animal labels and the continuous evidence score as a secondary measure of score separability.

Algorithm behavior was characterized using 60 candidate thresholds linearly spaced between the 10th and 99.5th percentiles of the animal training-set evidence distribution. At each threshold, event sensitivity and TTD were calculated from animal training recordings, while FP/hr was calculated from pediatric control recordings. After empirical review of the tradeoff among training-set sensitivity, detection latency, and patient-control false-alert burden, 3.0282 was manually fixed as the operating threshold. The fixed threshold was then evaluated exactly at that value and applied unchanged to the held-out animal test recordings and all subsequent analyses. Held-out animal recordings were not used during operating-point selection.

The threshold was applied to a unitless evidence score derived from locally normalized features rather than directly to raw voltage. It therefore represented a sustained departure from each recording’s local baseline rather than a fixed physical strain value. The same threshold could consequently be used across recordings while the rolling baseline and initial calibration accommodated differences in sensor placement, tissue compliance, and baseline signal characteristics.

### E. Animal Study:

An animal study (SW20M239) was conducted following initial approval (on 08/18/2020) by the Johns Hopkins University Animal Care and Use Committee (ACUC), ensuring compliance with institutional and regulatory standards for ethical animal research. Using piglets as the animal model, because porcine integumentary anatomy and physiology share important similarities with humans, controlled infusions were administered under general anesthesia [13]. A total of five piglets were used in the study, and each infusion type was administered on a separate limb, allowing one infusion per limb per animal. Each piglet was scheduled to receive infusions of normal saline, hypertonic saline (2–3%), warmed saline (37°C), and 10% calcium gluconate across separate limbs to simulate diverse clinical infiltration scenarios. Because calcium gluconate is a vesicant, its subcutaneous delivery technically modeled extravasation; however, infiltration is used throughout as an umbrella term for experimentally induced extravascular infusion events [14]. Infusions were initially delivered intravenously at a rate of 4 mL/kg/hour and subsequently redirected subcutaneously to realistically mimic infiltration conditions. Continuous measurements of skin strain and temperature were recorded to characterize infiltration events. Of the 20 planned limb-level recordings, four were excluded because reliable IV access could not be established, preventing acquisition of an adequate intravenous control. The analyzed animal dataset therefore comprised 16 recordings.

Data from this study were subsequently used to develop and empirically refine the rule-based detection algorithm. Specifically, the tests evaluated the speed and characteristics of skin swelling, sensor responsiveness to different infiltration phenotypes (localized versus diffuse), and the sensor’s ability to track swelling under controlled pump manipulations. Although a range of infusion types, including normal saline, hypertonic saline, warmed fluids, and calcium gluconate, were tested during the animal studies, the sample size for each condition was limited. Accordingly, infusion type was not incorporated as an input or stratification variable in algorithm development. This decision was intentional, as no grossly distinguishable or consistent differences in strain sensor signals were observed across infusion types. The primary motivation for testing multiple infusions was to evaluate whether distinct temperature profiles might emerge; however, no clinically meaningful or reproducible thermal changes were identified. As a result, the detection algorithm was designed to operate independently of infusion parameters, relying solely on strain-based signal dynamics normalized to each recording’s baseline.

Additionally, during the animal study, multiple infiltrations were observed and recorded from a single infusion in cases where no visible bump formed. These events were classified as diffuse infiltration phenotypes, characterized by circumferential or widespread fluid accumulation without a discrete elevation in the skin. In such cases, the sensor’s strain signal would return to initial baseline upon pausing the infusion pump, enabling subsequent re-infiltration events to be simulated by restarting the infusion. This approach provided additional positive events and enhanced the ability to evaluate the sensor’s responsiveness to subtle and diffuse infiltration profiles, which are more representative of early pediatric infiltrations.

### F. Early Clinical Testing

Following the successful completion of animal studies that demonstrated the sensitivity, feasibility, and preliminary safety of the strain-based infiltration detection device, we initiated a clinical pilot study at Johns Hopkins Hospital to begin evaluating the system in real-world pediatric settings. These human studies were launched after confirming low-volume infiltration detection without observed device-related adverse effects in the pig model and using those data to inform early algorithm development.

The clinical study, approved by the Johns Hopkins Medicine Institutional Review Board (IRB00305520, initially approved on 07/11/2022), was conducted in the PICU and perioperative pediatric settings. Eligible participants included hospitalized children with peripheral IV lines, including those undergoing surgery under the care of study-affiliated physicians. Written informed consent was obtained from a parent or legal guardian for all participants, and child assent was obtained when age and developmentally appropriate, in accordance with JHM IRB requirements and the Declaration of Helsinki. The study protocol did not alter standard clinical care; instead, the custom sensor was positioned approximately 2 cm from the IV catheter insertion site (needle entry point) and passively recorded continuous strain data without alerting clinicians or influencing treatment decisions.

When an infiltration is clinically diagnosed by the bedside nurse according to standard care protocols, the event is logged, and data collection from the device is stopped. These events are then used retrospectively to analyze strain signals and improve infiltration detection algorithms, with a specific focus on distinguishing infiltration-related changes from normal motion artifacts or other signal noise.

The primary long-term objective of this study was to compare the timing and accuracy of nurse-identified infiltration events to those retrospectively detectable by the strain sensor system. Secondary objectives include characterizing typical artifact profiles in the clinical setting and refining device thresholds for future real-time detection. No infiltration events were observed in the clinical cohort. Therefore, the pediatric recordings were not used to estimate event sensitivity or TTD. Instead, these control recordings were used to characterize real-world artifacts and baseline variability and to quantify FP/hr across candidate thresholds, which informed operating-point selection.

## III. Results

Intravenous infiltration detection performance was evaluated using 16 animal recordings, each containing at least one experimentally induced infiltration event. Several recordings included multiple distinct infiltration episodes within a single monitoring session, yielding a total of 35 infiltration events across all animal data. These repeated episodes enabled evaluation of continuous, event-level detection performance without recalibration. No device-related skin injury or other adverse effects were observed during animal testing. Following the animal study, five human inpatient recordings, totaling 117.8 hours of continuous monitoring without infiltration events, were used as control data to characterize false-positive alert burden under real-world clinical conditions and inform operating-point selection. A summary of the datasets used for algorithm development and evaluation is provided in Table 1.

**TABLE 1:** **Summary of datasets used for algorithm development and evaluation.** Animal recordings were divided at the file level into training and held-out test sets. Non-event duration represents monitoring time outside annotated infiltration events and was used as the denominator for calculating animal false alerts per hour (FP/hr). All pediatric control monitoring was non-event time because no clinical infiltrations occurred. Human control recordings were used to characterize false-alert burden and inform operating-point selection; they were not used to calculate sensitivity or TTD.

Dataset Type	Number of Files	Total Duration (hours)	Non-event Duration Used for FP/hr (h)	Number of Events	Purpose
Animal (Training)	11	3.51	1.627	26	Algorithm development and threshold selection
Animal (Test)	5	2.04	0.751	9	Held-out evaluation
Animal (Total)	16	5.55	2.379	35	Overall performance reporting
Human (Control)	5	117.8	117.8	0	False-alert characterization and operating point selection

Algorithm performance was assessed using an event-based evaluation framework, in which infiltration was treated as a continuous event rather than a pointwise classification problem. Clinically meaningful outcomes included event sensitivity, time-to-detection (TTD), and false-positive alert rate per hour (FP/hr). The primary performance objective was to balance early event detection and sensitivity against false-positive alert burden, reflecting the clinical importance of both timely detection and reduced alarm fatigue during continuous monitoring.

### A. Infiltration Phenotypes Observed in Animal Studies

Two distinct infiltration phenotypes were consistently observed during animal experiments, as illustrated in Fig. 5. The first and most common phenotype was a diffuse infiltration pattern (Fig. 5A), characterized by gradual, spatially distributed tissue expansion without formation of a discrete localized mass. Diffuse infiltrations allowed extravasated fluid to redistribute through compliant subcutaneous tissue, resulting in strain signals that increased during infusion and partially relaxed when infusion was paused. This behavior enabled multiple infiltration episodes to be induced and recorded within a single experiment, contributing substantially to the total number of evaluated events.

**FIGURE 5.**
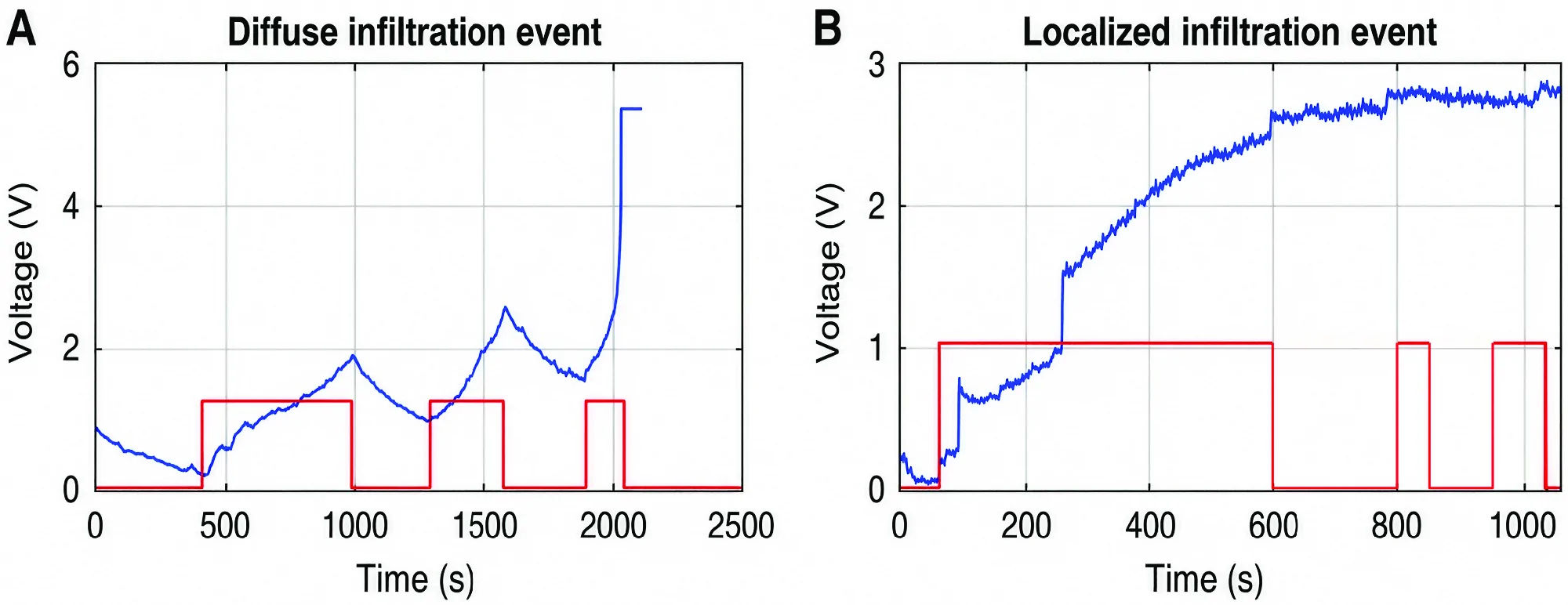
**Representative infiltration phenotypes observed during animal experiments.** The blue trace represents raw strain-sensor voltage, and the red trace indicates infusion pump status during experimentally induced infiltration (raised = pump on; baseline = pump off). The red trace is schematic and does not represent voltage. (A) Diffuse infiltration, characterized by spatially distributed tissue expansion without discrete localized swelling. The strain signal partially decreased during pump-off intervals and increased when pumping resumed, allowing repeated infiltration responses within a single experiment. (B) Localized “bump” infiltration, characterized by focal swelling near the IV insertion site. The strain signal remained elevated during pump-off intervals, including 600-800 and 850-950 seconds, consistent with persistent localized fluid accumulation. Pump interruptions therefore did not produce distinct, separated strain-response episodes in this phenotype.

The second phenotype was a localized “bump” infiltration (Fig. 5B), characterized by rapid formation of a focal swelling near the catheter insertion site. In these cases, extravasated fluid remained confined even after infusion was paused, producing a sustained strain signal that did not return toward baseline. Because localized swelling persisted, only a single infiltration episode could be recorded per experiment under this phenotype. Localized infiltrations were observed less frequently than diffuse infiltrations, consistent with the high tissue compliance and fluid dispersal capacity characteristic of juvenile-scale physiology.

The predominance of diffuse infiltration patterns has important implications for detection, as these events may not produce visually obvious localized swelling early in their course. The proposed system detected both diffuse and localized infiltration presentations within the animal dataset.

### B. Representative Trace

Fig. 6 presents a representative animal recording illustrating the relationship between the raw strain signal, the algorithm-generated evidence score, and the motion-related first difference signal during continuous monitoring. For clarity, only the first infiltration window is explicitly annotated in the figure; however, an additional infiltration episode is present later in the recording. In each case, sustained tissue deformation produces a corresponding rise in the evidence signal that crosses the alert threshold after infiltration onset while remaining below threshold during baseline periods. This example demonstrates both early detection during the initial event and reliable detection of subsequent infiltrations without restarting the algorithm or manually recalibrating the sensor, supporting the algorithm’s suitability for continuous bedside monitoring across repeated infiltration episodes.

**FIGURE 6.**
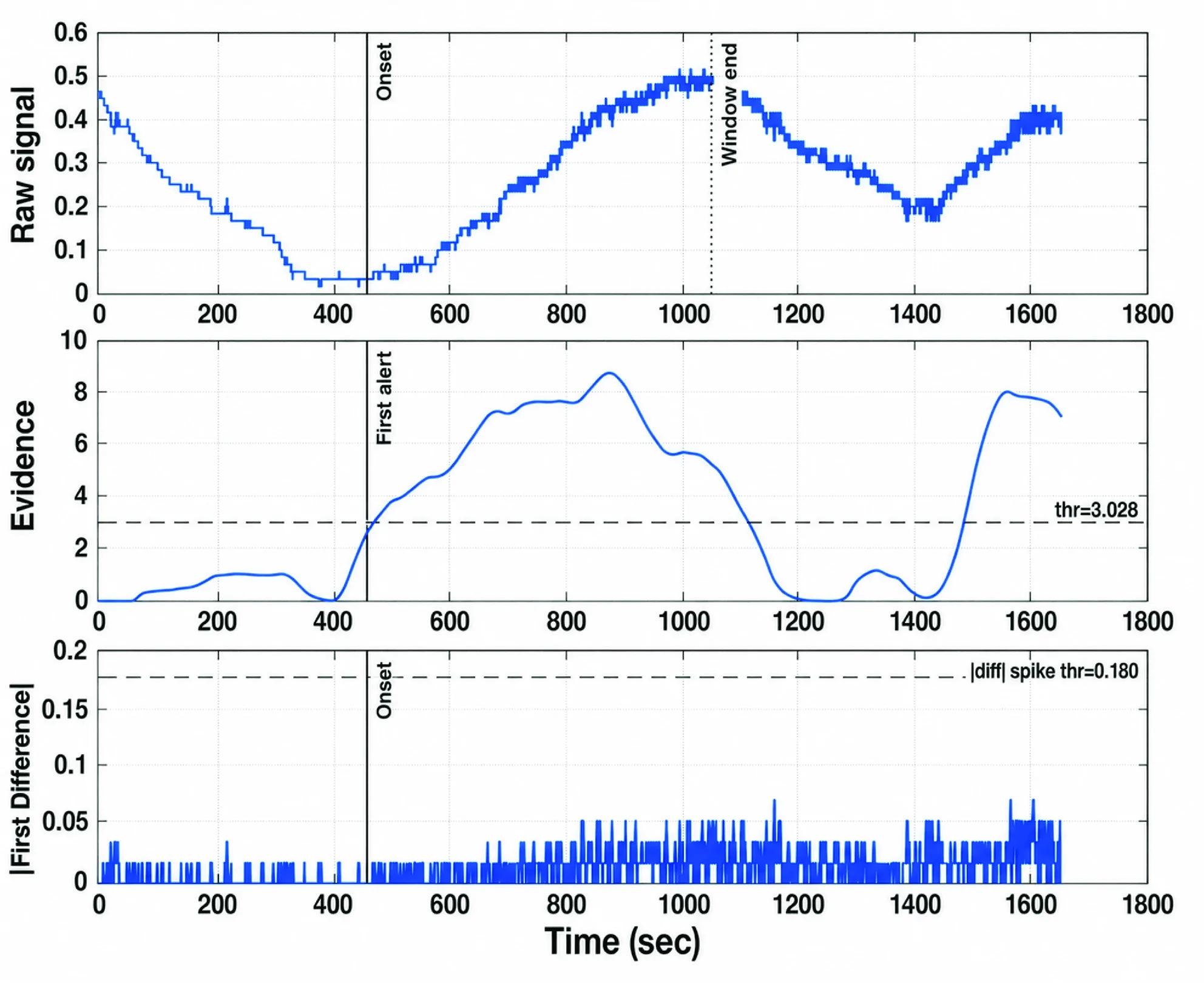
**Representative example of infiltration detection using the proposed real-time-capable algorithm.** Representative animal recording showing the raw strain signal, algorithm-generated detection evidence, and first difference-based motion veto. Vertical markers indicate the onset and end of the first annotated infiltration window, and the dashed line denotes the selected detection threshold (3.0282). Although only the first infiltration window is labeled for clarity, the evidence signal rises above threshold an additional time later in the recording, illustrating repeated event detection without restarting the algorithm.

### C. Time-to-Detection and Infused Volume at Detection

Across animal recordings, the algorithm achieved early detection of infiltration events at the selected operating threshold (3.0282), with median TTD values on the order of tens of seconds (Fig. 7). Across the full animal dataset, median TTD was 57.0 seconds, while mean TTD was 94.2 seconds, reflecting a small number of late detections that produced a long-tailed latency distribution. Approximately 86.7% of detected events occurred within the first two minutes of infiltration onset.

**FIGURE 7.**
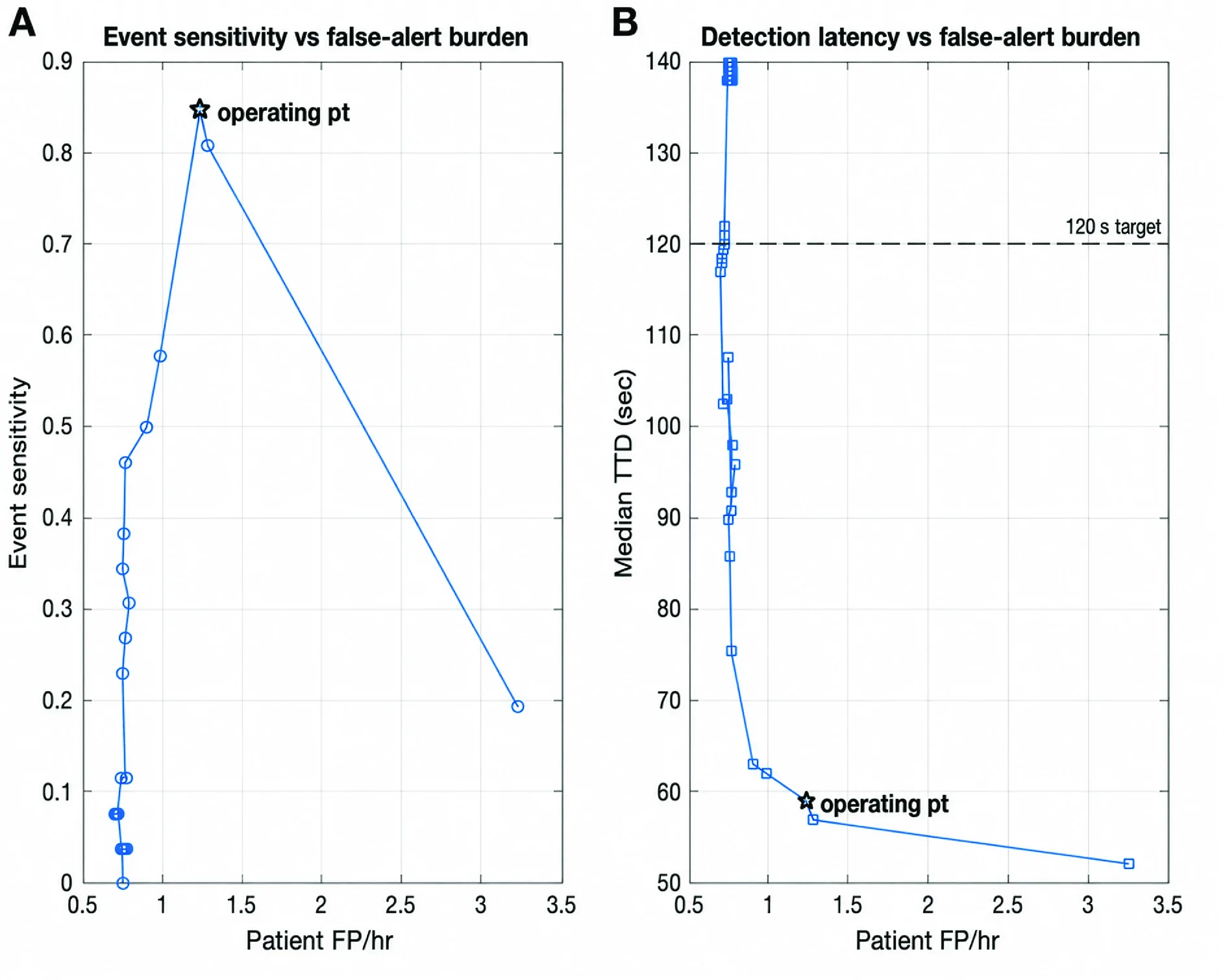
**Tradeoff between event sensitivity, detection latency, and false-positive alert burden.** Event-level detection performance evaluated across a range of detection thresholds using animal training recordings, with false-positive alert burden quantified using patient control data. **(A)** Event sensitivity (start-based; counted when the stored alert onset occurs between 10 seconds before the annotated event onset and the annotated event end) plotted as a function of patient FP/hr, illustrating the tradeoff between detection sensitivity and clinical alert burden during training-based operating-point selection. **(B)** Median time-to-detection (TTD) plotted as a function of patient FP/hr, with the dashed horizontal line indicating the selected 120-second early-detection target. The selected operating threshold is highlighted in both panels and represents a balance between high event sensitivity and low detection latency while maintaining a false-positive rate that is acceptable for prospective clinical evaluation.

Performance on the held-out test set was comparable. In the test subset, 8 of 9 infiltration events were detected (event sensitivity = 0.89), with a median TTD of 53.5 seconds. Mean TTD in the test set was higher (146.5 seconds) because of a small number of delayed detections, but 87.5% of detected test events occurred within the selected 120-second early-detection target.

A 120-second early-detection target was selected as a pragmatic benchmark for evaluating early-detection performance. This interval was chosen to represent a clinically useful opportunity for intervention before progression to overt swelling or significant tissue injury, rather than as a precise physiological injury threshold.

To contextualize detection latency in terms of infused volume, animal experiments were conducted at a standardized infusion rate of 4 mL/kg/hour. Assuming continuous leakage from infiltration onset, the observed median TTD of 57.0 seconds corresponds to an estimated infused volume of approximately 0.063 mL/kg, while 120 seconds corresponds to approximately 0.133 mL/kg. For a 10-kg subject, these values correspond to approximately 0.63 mL and 1.33 mL, respectively. For this 10-kg example, both values are below 2 mL and would generally be expected to precede visually obvious swelling or significant tissue injury.

Diffuse infiltrations, which represented the majority of observed events, often produced subtle, spatially distributed tissue expansion rather than discrete localized swelling. Detection within this low-volume range indicates sensitivity to early physiological changes before development of clinically obvious swelling.

### D. Event-Level Detection Performance

Event-level detection performance across animal recordings is summarized in Fig. 7, which reflects a training-set threshold sweep used for operating-point selection, illustrating the tradeoff between event sensitivity, detection latency, and false-positive alert burden across a range of detection thresholds. At very low thresholds, frequent false alerts can cause alert episodes to begin more than 10 seconds before an annotated infiltration onset. Under the start-based criterion, these episodes are not credited as event detections even if they subsequently overlap the event, which can produce non-monotonic points in the sensitivity–FP/hr tradeoff (rightmost point in Fig. 7A). At the manually fixed operating threshold of 3.0282, the algorithm detected 30 of 35 infiltration events across all animal data (event sensitivity = 0.86), with event sensitivity of 0.85 in the training set and 0.89 in the held-out test set.

Detection performance was evaluated across individual animals (Table 2), with event-level sensitivity ranging from 0.71 to 1.00 across subjects. Most animals demonstrated high sensitivity ( *≥ 0.88*). Differences in sensor placement, infiltration phenotype, tissue response, and other setup-related factors inherent to early-stage animal experiments may have contributed to between-animal variability.

**TABLE 2:** **Per-animal event-level detection performance.** Sensitivity is reported as the proportion of detected infiltration events per subject.

Animal	Total Events	Detected Events	Event Sensitivity
Animal 1	7	5	0.71
Animal 2	4	3	0.75
Animal 3	9	8	0.89
Animal 4	3	3	1.00
Animal 5	12	11	0.92

Fig. 8 presents performance on the held-out test dataset alongside the full animal-dataset summary for descriptive context (test: 8 of 9 events detected; full dataset: 30 of 35 events detected).

**FIGURE 8.**
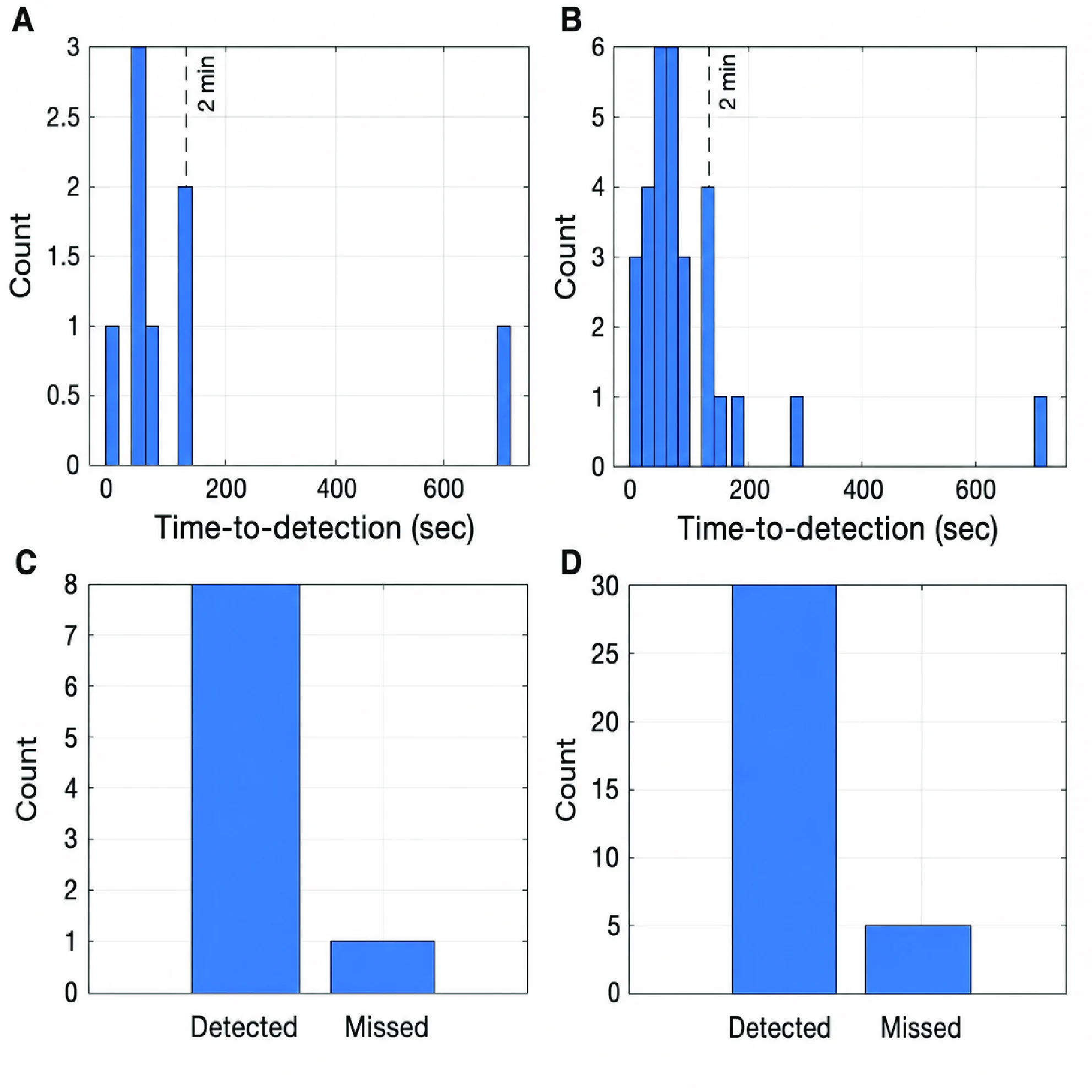
**Comparison of event-level detection performance on held-out test data and the full animal dataset.** Event-level detection results evaluated at the selected operating threshold (thr = 3.0282). **(A)** Time-to-detection (TTD) distribution for detected infiltration events in the held-out test dataset, with the dashed vertical line indicating the 120-second early-detection target. **(B)** TTD distribution for detected infiltration events across the full animal dataset. **(C)** Detected versus missed infiltration event counts in the test dataset (8 detected, 1 missed). **(D)** Detected versus missed infiltration event counts across all animal recordings (30 detected, 5 missed). The held-out results were directionally similar to the full-dataset summary.

As a secondary descriptor of score separability, pooled pointwise ROC AUC values were 0.72 for training data, 0.70 for test data, and 0.71 across all animal data. Due to extreme class imbalance and the continuous monitoring context, ROC AUC was not used for threshold selection, and interpretation focused on event-based performance metrics.

### E. False-Positive Alert Burden in Clinical Control Data

False-positive alert burden was quantified using both animal non-event periods and human inpatient control recordings. The pediatric control dataset comprised 117.8 hours of continuous monitoring without infiltration events and was used to characterize false-alert burden under real-world clinical conditions involving patient motion, nursing interaction, and environmental noise. Because these recordings also informed operating-point selection, the reported patient-control FP/hr represents a development-stage estimate rather than an independent held-out clinical validation result.

At the fixed threshold of 3.0282, the pediatric control recordings produced 146 false-alert episodes over 117.8 hours of non-event monitoring, corresponding to 1.239 FP/hr. The held-out animal test recordings produced two false-alert episodes over 0.751 hours of non-event monitoring, corresponding to 2.662 FP/hr and an event-level precision of 0.800. Across all animal recordings, five false-alert episodes over 2.379 hours of non-event monitoring corresponded to 2.102 FP/hr and an event-level precision of 0.857. This level of false-positive alerting was considered acceptable for feasibility assessment in a controlled clinical research setting, while acknowledging that further reduction would be necessary for routine clinical deployment. Threshold sweeps identified neighboring operating points with reduced alert burden at the cost of lower event sensitivity and longer detection latency (Fig. 7), illustrating the tradeoff between early detection and alarm burden.

## IV. Discussion

### A. Overall Algorithm Performance

This study demonstrates the feasibility of using a custom strain-sensing patch, mounted with standard clinical dressings, and a real-time-capable detection framework to identify IV infiltration events during continuous monitoring. Using an event-based evaluation framework, the proposed system achieved reliable detection of infiltration events across controlled animal experiments, while maintaining a false-positive rate that is acceptable for prospective clinical evaluation when evaluated on real-world patient control data.

Although the algorithm is designed for real-time deployment, performance in this study was evaluated retrospectively using recorded datasets. As a result, practical considerations associated with true real-time implementation, including hardware constraints, data transmission latency, and integration with clinical monitoring systems, were not directly assessed. Future work will include full hardware-integrated validation to confirm real-time performance under clinical operating conditions.

At the selected operating threshold (3.0282), the algorithm achieved an event sensitivity of 0.85 on the training dataset and 0.86 across the full animal dataset, with a median TTD of 57 seconds and a mean detection latency of approximately 94 seconds. Approximately 87% of detected events occurred within the selected 120-second early-detection target. Performance was similar on the held-out recording subset, where 8 of 9 infiltration events were detected with comparable TTD outcomes.

Importantly, algorithm performance was achieved using strictly causal signal processing and persistence-based alert logic, reflecting realistic deployment constraints for bedside monitoring systems.

### B. Clinical Implications of Early Detection

The observed detection latencies have meaningful clinical implications, particularly in high-acuity environments such as the NICU and PICU. In the animal model, median detection occurred in 57 seconds, substantially earlier than routine visual assessment would be expected to identify the low-volume tissue changes observed in this study.

When contextualized by infusion rate, early detection corresponded to very small estimated infused volumes at the time of alert. Assuming continuous leakage from infiltration onset, the median TTD of 57 seconds at 4 mL/kg/hour corresponds to approximately 0.063 mL/kg, while the 120-second target corresponds to approximately 0.133 mL/kg. For a 10-kg subject, these values correspond to approximately 0.63 mL and 1.33 mL, respectively. Both are below the approximately 2-mL average detection volume reported for ivWatch [7] and below volumes generally associated with overt swelling or significant tissue injury. Detection within this range could therefore provide an opportunity for intervention before progression to more consequential tissue damage.

### C. Advantages Over Existing Solutions

Compared to existing commercial IV infiltration detection systems that rely on optical sensing and proprietary hardware, the proposed strain-based approach offers several practical and economic advantages. The sensing system is constructed from low-cost, widely available components, including off-the-shelf resistive materials and standard medical-grade Tegaderm dressings that are already approved for routine clinical use. This eliminates the need for custom optics, specialized adhesives, or rigid sensor housings, reducing both manufacturing cost and barriers to clinical adoption.

The sensor design allows the electronics to remain mechanically isolated from direct skin contact, relying instead on strain propagation through a soft, conformable dressing. This improves patient safety and comfort while maintaining mechanical compliance with pediatric anatomy. Together, these design choices support scalability and potential deployment in resource-limited settings, community hospitals, or outpatient environments where existing commercial systems may be impractical due to cost or complexity.

In addition to its simplified sensing approach, the proposed system was designed around a reusable electronic reader and a low-cost disposable sensing interface. The reusable reader contains the principal sensing, control, and communication architecture required for continuous monitoring, including a differential amplifier, microcontroller, and Bluetooth communication module. These components have a preliminary low-volume engineering bill of materials of approximately *$*30. In this configuration, the system could operate as a complete monitoring platform when a Bluetooth-connected phone, tablet, or workstation serves as the user interface and primary alerting device.

The existing microcontroller is also capable of controlling low-cost peripheral outputs, including indicator lights, an audible alarm, user controls, and a display, without requiring modification of the underlying sensing architecture. If a dedicated onboard dashboard and user interface were desired, these peripheral components, together with an associated power supply, enclosure, and mounting hardware, are preliminarily estimated to add approximately *$*50. A fully self-contained bedside implementation would therefore have an estimated low-volume engineering bill of materials of approximately *$*80. These values are engineering estimates rather than anticipated commercial selling prices and exclude manufacturing, validation, regulatory compliance, technical support, and distribution.

The disposable sensing interface consists of two transparent medical dressings and a small amount of piezoresistive material. Although the piezoresistive sensing material itself contributes only cents to the cost of each patch, the complete prototype disposable has an estimated material cost of approximately *$*0.50– *$*1.50 per monitored IV site, depending on dressing size and purchasing volume. This estimate excludes assembly, electrical connections, packaging, quality-control testing, regulatory compliance, and distribution. Therefore, the eventual commercial price would be higher than the prototype material cost.

Like the proposed system, ivWatch uses a reusable monitoring component together with a disposable sensor applied to each monitored IV site. The most clearly quantifiable economic difference is the recurring cost of the disposable interface. The manufacturer identifies the proprietary SmartTouch sensor as a single-use product [15], and a current public secondary-market listing advertises a new sensor at an asking price of *$*47.50 per unit, although actual institutional pricing may differ [16]. In comparison, the complete prototype material cost of the proposed disposable patch is estimated at approximately *$*0.50– *$*1.50 per monitored IV site. The publicly listed SmartTouch asking price is therefore approximately 32–95 times the proposed patch’s estimated prototype material cost. This is not a direct comparison between final commercial selling prices because the proposed estimate excludes assembly, packaging, quality control, regulatory compliance, distribution, and commercial markup. Nevertheless, the magnitude of the difference demonstrates the potential for the proposed commodity-material architecture to substantially reduce the recurring cost of continuous monitoring.

ivWatch also requires a specialized proprietary patient monitor. A publicly verifiable manufacturer or institutional price for this monitor was not identified; therefore, no specific ivWatch capital cost is assigned. Nevertheless, the requirement for dedicated commercial monitoring hardware introduces a separate capital purchase and may also involve service, support, and procurement costs. By contrast, the proposed system’s *$*30 reusable reader could use an existing Bluetooth-connected device as its alert and user interface, potentially avoiding the need for a dedicated monitor. If a self-contained bedside interface were preferred, the existing controller architecture could support the required display, audible and visual alarms, and user controls using the additional low-cost peripheral hardware described above. Although these engineering estimates cannot be directly compared with a final commercial ivWatch selling price, the proposed architecture provides flexibility that may reduce both recurring disposable costs and the need for costly specialized capital equipment.

### D. Infiltration Phenotypes and Implications for Detection

The majority of infiltration events involved gradual, spatially distributed subcutaneous fluid accumulation rather than rapid formation of a discrete localized swelling. Only a minority of experiments exhibited a focal “bump” phenotype, in which extravasated fluid remained localized and strain did not return toward baseline after infusion was paused.

Diffuse infiltrations are particularly challenging to detect visually and may not generate sufficient localized pressure to trigger point-based or contact-dependent sensors early in their course. By contrast, the proposed sensor measures deformation of the dressing itself, enabling detection of subtle, distributed strain patterns that precede obvious clinical signs. The system’s detection of both diffuse and localized profiles demonstrates performance across these two presentations within the animal dataset.

### E. Differentiation of Infiltration Signals from Motion

A key requirement for continuous IV monitoring is the ability to distinguish true infiltration-related deformation from routine motion artifacts caused by patient movement, nursing interaction, or environmental disturbances. In this study, human inpatient recordings totaling 117.8 hours were used to characterize false-positive alert burden under real-world conditions, despite the absence of infiltration events.

At the selected operating threshold, the algorithm produced approximately 1.2 false alerts per hour in patient recordings. When evaluated over non-event periods within the held-out animal recordings, the corresponding false-positive rate was approximately 2.66 alerts per hour (2 alerts over 0.751 hours). Threshold sweeps further identified neighboring operating points with reduced alert burden at the cost of lower event sensitivity and longer detection latency. This demonstrates that the algorithm can be tuned to prioritize either early detection or reduced alert burden depending on clinical context.

The clinical significance of this alert rate must be interpreted in the context of the intended stage of system development. At approximately 1.2 false alerts per hour during the pediatric clinical monitoring, the observed rate is higher than would likely be desirable for routine clinical deployment, where minimizing alarm fatigue is critical. However, this level of alerting is sufficiently low to permit feasible prospective clinical evaluation, particularly in a controlled research setting where alerts can be assessed without directly influencing clinical care. Importantly, the system is designed with tunable operating thresholds, allowing adjustment of the tradeoff between early detection sensitivity and false alert burden depending on the clinical context. Future work will focus on reducing false-positive alerts through both algorithmic refinement and hardware improvements prior to real-world deployment.

The current datasets separately characterized detection of induced animal infiltrations and false-alert behavior during routine pediatric clinical monitoring. Infiltrations occurring concurrently with significant motion were not observed and remain an open challenge. Additional data will be required to characterize performance in these more complex scenarios.

### F. Future Hardware Optimization

Future work will prioritize hardware-side refinements aimed at reducing the introduction of irrelevant strain signals prior to algorithmic processing. Rather than relying solely on post hoc signal filtering, planned improvements include modifications to sensor geometry, mounting configuration, and mechanical isolation to better decouple infiltration-related tissue deformation from limb movement or external disturbances. Improving signal fidelity at the hardware level is expected to further reduce false-positive alerts and improve robustness in high-motion clinical environments.

Future work may also explore machine learning-based approaches for infiltration detection, provided that such methods can be implemented with low-latency performance suitable for real-time operation and without substantially increasing computational burden, memory requirements, or the cost and complexity of the embedded hardware.

### G. Role of Temperature Sensing

Although temperature sensing is commonly cited as an early indicator of IV infiltration and is frequently relied upon by bedside clinicians, thermistor data collected during animal experiments did not demonstrate consistent or discriminative changes during infiltration events in this study. This likely reflects the short duration of the experiments, which were designed to rapidly induce infiltration and evaluate early detection, limiting the development of inflammatory temperature gradients.

As a result, temperature measurements were excluded from the final detection algorithm. Longer-duration monitoring in future clinical studies may reveal scenarios in which temperature differentials serve as a useful secondary confirmation signal, but strain-based sensing provided the most reliable early indicator in the present dataset.

### H. Regulatory and Translational Considerations

From a translational perspective, the proposed system is currently in an early-stage feasibility and validation phase and has not yet undergone regulatory review or approval. In the present clinical study, the device was used only for passive monitoring and did not generate clinical alerts or alter patient care. Future clinical deployment would require regulatory clearance or approval as a medical device, supported by prospective clinical validation demonstrating safety, reliability, usability, and clinical benefit. In the United States, this type of non-invasive bedside monitoring system would likely require evaluation through an FDA medical device pathway, such as a Class II 510(k) or De Novo pathway depending on predicate device availability and final intended use. More broadly, investigators seeking to translate similar systems should engage their institutional regulatory teams and relevant regulatory authorities early to define device classification, risk controls, validation requirements, and clinical evidence needed for deployment.

### I. Limitations and Generalizability

This study has several limitations related to generalizability and stage of clinical validation. The detection algorithm was primarily developed and evaluated using controlled animal experiments, and although human inpatient recordings were included to characterize false-positive alert burden, no clinical infiltration events were observed during the initial patient study phase. As a result, the ability of the system to detect spontaneous infiltration events in human patients remains unproven.

Animal recordings were partitioned at the file level rather than grouped by animal. Accordingly, the held-out analysis assessed performance on held-out recordings but did not constitute fully independent subject-level validation. In addition, because the pediatric control recordings informed operating-point selection, the reported patient-control FP/hr is a development-stage estimate rather than an independent held-out clinical validation result.

In addition, there are important differences between the controlled animal model and real-world clinical conditions. Infiltrations in anesthetized piglets were experimentally induced under controlled infusion conditions, whereas human infiltrations occur spontaneously and may be influenced by patient movement, variable catheter positioning, and heterogeneous tissue properties. These factors may alter the temporal and spatial characteristics of tissue deformation and could impact detection performance.

Despite these limitations, the current study was designed as a feasibility and early validation effort to establish sensitivity to infiltration-related strain signals and characterize false-positive behavior under real clinical noise conditions. The inclusion of extended-duration human monitoring data without infiltration events provides valuable insight into system behavior in the presence of motion artifacts and routine clinical activity.

Future work will focus on prospective clinical validation in patient populations with confirmed infiltration events, enabling direct assessment of detection sensitivity, time-to-detection, and alert reliability under real-world conditions.

## V. Conclusion

This study presents a novel, skin-safe, and cost-effective IV infiltration detection system that combines a custom strain-sensing patch with a real-time-capable, event-based detection algorithm. In controlled animal experiments, the system achieved early detection of infiltration events with a median TTD under one minute, corresponding to approximately 0.063 mL/kg of infused volume at the experimental flow rate.

By enabling detection of diffuse, spatially distributed tissue deformation that may precede visually apparent swelling, the proposed approach addresses a critical limitation of existing IV monitoring strategies. While further clinical validation is required, particularly in noisy or high-motion scenarios, these results support the feasibility of continuous IV infiltration monitoring in pediatric and other low-flow infusion settings. With continued hardware optimization and expanded clinical testing, this system has the potential to improve patient safety while reducing the burden of manual IV site surveillance.

## Data Availability

The datasets generated and analyzed during the current study are available from the corresponding author upon reasonable request.

## Conflicts of Interest

T. El-Khalili and R. Yang are co-inventors on patent WO 2023/205697 A2 related to the technology described in this work. The remaining authors declare no competing interests.

## References

[1] L. Dougherty, “IV therapy: Recognizing the differences between infiltration and extravasation,” Brit. J. Nursing, vol. 17, no. 14, pp. 896–901, Jul. 2008, doi: 10.12968/bjon.2008.17.14.30656. 18935841

[2] S. J. Phelps and R. A. Helms, “Risk factors affecting infiltration of peripheral venous lines in infants,” J. Pediatrics, vol. 111, no. 3, pp. 384–389, Sep. 1987, doi: 10.1016/s0022-3476(87)80458-4. 3625405

[3] S. M. Park, I. S. Jeong, and S. S. Jun, “Identification of risk factors for intravenous infiltration among hospitalized children: A retrospective study,” PLoS ONE, vol. 11, no. 6, Jun. 2016, Art. no. e0158045, doi: 10.1371/journal.pone.0158045. PMC4924835 27351488

[4] D. Doellman, “Infiltration and extravasation: Update on prevention and management,” J. Infusion Nursing, vol. 32, no. 4, pp. 203–211, 2009, doi: 10.1097/nan.0b013e3181aac042. 19605999

[5] L. Hadaway, “Infiltration and extravasation,” AJN, Amer. J. Nursing, vol. 107, no. 8, pp. 64–72, Aug. 2007, doi: 10.1097/01.naj.0000282299.03441.c7. 17667395

[6] J. A. Jambulingam, R. McCrory, L. West, and O. T. Inan, “Non-invasive, multi-modal sensing of skin stretch and bioimpedance for detecting infiltration during intravenous therapy,” in Proc. 38th Annu. Int. Conf. IEEE Eng. Med. Biol. Soc. (EMBC), Orlando, FL, USA, Aug. 2016, pp. 4755–4758, doi: 10.1109/EMBC.2016.7591790. 28269333

[7] ivWatch. (Jul. 7, 2020). IvWatch SmartTouch Sensor Receives FDA Clearance for Continuous IV Site Monitoring. PR Newswire. Accessed: Jan. 9, 2026. [Online]. Available: https://www.prnewswire.com/news-releases/ivwatch-smarttouch-sensor-receives-fda-clearance-for-continuous-iv-site-monitoring-301089088.html

[8] A. O. Bicen, L. L. West, L. Cesar, and O. T. Inan, “Toward non-invasive and automatic intravenous infiltration detection: Evaluation of bioimpedance and skin strain in a pig model,” IEEE J. Translational Eng. Health Med., vol. 6, pp. 1–7, 2018, doi: 10.1109/JTEHM.2018.2815539. PMC5912429 29692956

[9] J.-H. Kim, B.-J. Shin, S.-W. Baik, and G.-R. Jeon, “Early detection of intravenous infiltration using multi-frequency bioelectrical impedance parameters: Pilot study,” J. Sensor Sci. Technol., vol. 26, no. 1, pp. 15–23, Jan. 2017, doi: 10.5369/jsst.2017.26.1.15.

[10] D. Wang, H. Xu, S. Chen, X. Lou, J. Tan, and Y. Xu, “Medical adhesive-related skin injuries and associated risk factors in a pediatric intensive care unit,” Adv. Skin Wound Care, vol. 32, no. 4, pp. 176–182, Apr. 2019, doi: 10.1097/01.asw.0000553601.05196.fb. 30845071

[11] S. Kamada, R. Mosier, T. El-Khalili, S. Triantis, and R. Yang, “Scoping review of early intravenous infiltration and extravasation detection devices,” J. Infusion Nursing, vol. 46, no. 2, pp. 97–106, 2023, doi: 10.1097/nan.0000000000000499. 36853872

[12] R. Yang, T. El-Khalili, S. Kamada, and R. Mosier, “Device for early detection of pediatric IV infiltration,” WO Patent 2023/205 697 A2, Oct. 26, 2023.

[13] M. M. Swindle, A. Makin, A. J. Herron, F. J. Clubb, and K. S. Frazier, “Swine as models in biomedical research and toxicology testing,” Veterinary Pathol., vol. 49, no. 2, pp. 344–356, Mar. 2012, doi: 10.1177/0300985811402846. 21441112

[14] F. J. Pacheco Compaña, J. Midón Míguez, and F. J. de Toro Santos, “Lesions associated with calcium gluconate extravasation: Presentation of 5 clinical cases and analysis of cases published,” Ann. Plastic Surg., vol. 79, no. 5, pp. 444–449, Nov. 2017, doi: 10.1097/sap.0000000000001110. 28570460

[15] ivWatch. SmartTouch Sensors. Accessed: Jul. 20, 2026. [Online]. Available: https://www.ivwatch.com/sensors/

[16] DOTmed. IvWatch AC-10010003 SmartTouch Sensor. Accessed: Jul. 20, 2026. [Online]. Available: https://www.dotmed.com/listing/disposables-general/ivwatch/ac-10010003/iv-watch-smart-touch-sensor/5503490

